# An intronic SNP affects skeletal muscle development by regulating the expression of *TP63*

**DOI:** 10.3389/fvets.2024.1396766

**Published:** 2024-06-12

**Authors:** Yufen Chen, Zhen Wang, Xiaolu Qu, Bangmin Song, Yueting Tang, Bugao Li, Guoqing Cao, Guoqiang Yi

**Affiliations:** ^1^Guangdong Laboratory of Lingnan Modern Agriculture, Key Laboratory of Livestock and Poultry Multi-omics of MARA, Agricultural Genomics Institute at Shenzhen, Chinese Academy of Agricultural Sciences, Shenzhen, China; ^2^College of Animal Science, Shanxi Agricultural University, Jinzhong, China; ^3^Kunpeng Institute of Modern Agriculture at Foshan, Chinese Academy of Agricultural Sciences, Foshan, China; ^4^Bama Yao Autonomous County Rural Revitalization Research Institute, Bama, China

**Keywords:** TP63, myoblasts, proliferation, allelic variation, MEF2A

## Abstract

**Background:**

Porcine skeletal muscle development is pivotal for improving meat production. *TP63*, a transcription factor, regulates vital cellular processes, yet its role in skeletal muscle proliferation is unclear.

**Methods:**

The effects of *TP63* on skeletal muscle cell viability and proliferation were investigated using both mouse and porcine skeletal muscle myoblasts. Selective sweep analysis in Western pigs identified *TP63* as a potential candidate gene for skeletal muscle development. The correlation between TP63 overexpression and cell proliferation was assessed using quantitative real-time PCR (RT-qPCR) and 5-ethynyl-2’-deoxyuridine (EDU).

**Results:**

The study revealed a positive correlation between *TP63* overexpression and skeletal muscle cell proliferation. Bioinformatics analysis predicted an interaction between MEF2A, another transcription factor, and the mutation site of *TP63*. Experimental validation through dual-luciferase assays confirmed that a candidate enhancer SNP could influence MEF2A binding, subsequently regulating *TP63* expression and promoting skeletal muscle cell proliferation.

**Conclusion:**

These findings offer experimental evidence for further exploration of skeletal muscle development mechanisms and the advancement of genetic breeding strategies aimed at improving meat production traits.

## 1 Introduction

The meat production capacity of pigs is closely linked to the development of skeletal muscle, which serves as the primary source of animal protein (1, 2). Skeletal muscle mass accounts for a significant proportion of body weight, emphasizing the importance of muscle cell differentiation and proliferation in enhancing pig growth rate (3–5). Myogenesis is a coordinated process involving various stages, including the identification of multipotential mesodermal cells, proliferation and migration of adult myoblasts, fusion of myoblasts into multinucleated muscle fibers, and maturation of muscle fibers (6–8). Therefore, studying the molecular mechanisms underlying porcine skeletal muscle growth and development is essential to improve the growth rate and yield of pork (9, 10).

Skeletal muscle formation and meat production traits are regulated by intricate molecular mechanisms that involve the interplay of various genes and pathways (11, 12). Extensive research has been conducted around the world, resulting in significant progress and the identification of several genes and molecular markers associated with meat production traits (13, 14). For example, many QTL-related studies found that the selected genes of local pigs in China were mainly related to fat and reproduction traits, while the selected genes of lean pork breeds were enriched in body weight, growth rate, and carcass traits (15–18). With the implementation of high-throughput genotyping techniques, identifying selective sweeps at the genome level has become possible (19). Rubin et al. identified three genes (*NR6A1*, *PLAG1*, and *LCORL*) that contribute to the body length of European domesticated pigs (20). Ma et al. revealed strong signatures of selection in Duroc that can affect lean muscle mass (21, 22). However, a deeper understanding of the underlying molecular mechanisms is necessary to optimize breeding strategies and enhance meat quality.

TP63 and TP73, its counterpart, encompass multiple isoforms that independently regulate various genes or interact with other families of transcription factors (23). For instance, TAp63α has been demonstrated to promote proliferation in the mouse epidermis (24, 25). TP63 is a conserved transcription factor with multiple binding sites distributed throughout the genome (26). By directly binding to gene promoters, TP63 exerts control over downstream gene expression (27). Notably, TP63 has been implicated in myogenesis and muscle contraction, resulting in significant alterations in the expression of myogenic differentiation genes such as MYH9, MYH10, and CDKN1A (28). However, the precise role of TP63, in skeletal muscle development remains elusive (29).

This study aimed to investigate the molecular mechanisms by which TP63 regulates skeletal muscle development. First, we characterized the expression patterns of TP63 through the skeletal muscle transcriptome at 27 developmental time points, and investigated the function of TP63 in skeletal muscle development through knockdown and overexpression. The JASPAR database was used to predict the transcription factor motifs bound by TP63 mutation sites, and the molecular mechanism of SNP-binding transcription factors MEF2A regulating skeletal muscle development was explored. This study provides a theoretical basis for improving meat production performance and enhancing genetic improvement in pigs.

## 2 Materials and methods

### 2.1 Cell culture

Porcine skeletal muscle cells (BIOSPECIES-0017a) were purchased from Guangzhou Suyan Biotechnology Co., Ltd., Guangdong, China. C2C12 myoblasts and 293 T cells were purchased from the American Type Culture Collection (ATCC). The growth medium was Dulbecco’s Modified Eagle’s Medium (DMEM, Corning, China). contained 10% fetal bovine serum (FBS, Gibco, California, USA) and 1% penicillin–streptomycin (PS, Thermo Scientific, Massachusetts, USA). Cells were then placed in a 37°C cell incubator containing 5% oxygen and 95% carbon dioxide.

### 2.2 Skeletal muscle collection

Longissimus dorsi muscle samples were meticulously collected from Landrace and Tongcheng pigs across a comprehensive spectrum of 27 developmental time points. These animals were granted unrestricted access to food and water, and they were uniformly housed under controlled conditions to minimize environmental variations. Notably, at each time point, tissue samples were meticulously harvested from three distinct pigs to ensure robust biological replication. Following collection, all specimens were promptly flash-frozen in liquid nitrogen and preserved until subsequent RNA-seq analyses (GSE157045 and PRJNA754250) (18, 30).

### 2.3 RNA interference and overexpression

For RNA interference, negative control siRNA (siRNA-NC) and mouse siRNA-*TP63* were purchased from Gemma Pharmaceutical Technology (Shanghai, China). The sequences of the *TP63*-targeted siRNAs are listed in Supplementary Table S1. The *TP63* expression vector (pcDNA3.1-*TP63*) was synthesized by Gene Create (Wuhan, China). The control plasmid pcDNA3.1 was obtained from our laboratory. The coding sequences (CDSs) of the porcine *TP63* gene were inserted into the PLV3 vector (Invitrogen). Porcine skeletal muscle cells and C2C12 cells were inoculated into 6 or 12 well plates 12 h before treatment and then transfected with siRNA or plasmid using Attractene transfection reagent (Qiagen) according to its instructions. The small interfering RNA (siRNA) and plasmid were transfected at a final concentration of 50 nM. This precise concentration was selected following thorough optimization experiments, aligning with the widely reported effective range documented in the existing literature for proficient gene knockdown.

### 2.4 RNA extraction and real-time quantitative PCR (qPCR)

Cells or tissues were lysed with Triol (Invitrogen, Shanghai, China), chloroform, and denatured with isopropanol to precipitate RNA, then washed with 75 and 100% ethanol, respectively, and finally solubilized in DEPC water. RNA quality was determined by NanoDrop 2000 (Thermo Fisher Scientific, Massachusetts, USA). The identified RNAs were available for further studies. HiScript III First Strand cDNA Synthesis Kit (+gDNA wiper) (R312-01, Vazyme, Nanjing, China) was used for cDNA reverse transcription synthesis of mRNA, respectively, according to the instructions. In addition, Taq Pro Universal SYBR qPCR Master Mix (Vazyme, Nanjing, China) was used in the total reaction for messenger RNA qPCR in a volume of 20 μL, including 10 μL 2× SYBR Master Mix, 0.4 μL PCR forward primer, 0.4 μL polymerase Chain Reaction Reverse Primer, 2 μL cDNA, and 7.2 μL sterile enzyme-free water. Reaction conditions were 95°C for 30 s, followed by 95°C for 10 s and 65°C for 30 s for 40 cycles; this reference gene was Gapdh. Relative expression levels of messenger ribonucleic acid (RNA) were analyzed by the 2-∆∆CT method. Sequence information of the primers used for reverse transcription and quantification (Sangon Biotech, Shanghai, China) is shown in Supplementary Tables S2, S3.

### 2.5 5-ethynyl-2‘-deoxyuridine assay (EdU)

EdU assay was performed using EdU assay kit (Beyotime, China) and cell proliferation ability was determined using EdU-488 cell proliferation assay kit (Beyotime, China). Cells were spread onto 6-well plates, which were disrupted and overexpressed, and the final concentration of EdU was adjusted to 10% μm was added to each well, and incubation was continued for 1–2 h in the cell incubator. Cells were washed three times with PBS (Thermo Fisher) and then fixed with 4% formaldehyde fixative for 15 min at room temperature. The fixative was removed and the cells were washed three times with detergent. 1 mL of 0.3% Triton X-100 diluted in PBS was added and incubated at room temperature for 15 min to increase cell membrane permeability. In addition, 500 μL of the prepared click reaction solution was added to each well, and the cells were incubated for 30 min at room temperature and protected from light to observe and quantify the number of EdU-stained cells, and the nuclei were stained with DAPI (1:1000 PBS). Three fields were randomly selected for statistical analysis.

### 2.6 Cell counting kit-8 proliferation assay

C2C12 adult myoblasts and skeletal myoblasts were inoculated into 96-well plates and harvested at 0 h, 24 h, 36 h, 48 h, and 72 h post-transfection, respectively. The proliferation of adult myocytes was measured using Cell Counting Kit-8 (CCK-8) (Beyotime C0038, Beijing, China). A 1:9 mixture of CCK-8 reagent and complete culture medium was added to 96-well plates and continued at 37°C for 1 h. The cells were counted using a microplate reader. The optical density (OD) at 450 nm of each sample was measured with a microplate reader and growth curves were plotted.

### 2.7 Cell cycle assay

Skeletal muscle cells were spread into 6-well plates, disrupted and overexpressed, and collected after 48 h. The cells were digested with trypsin, centrifuged at 1000 g for 3–5 min, precipitated, and single-cell suspensions were prepared. The cells were washed with pre-cooled PBS and centrifuged again. Fix the cells, resuspend the cells with 1 mL of pre-cooled 70% ethanol, and resuspend at 4°C for 2 h or overnight. An appropriate amount of propidium iodide (PI) staining solution was prepared according to the Cell Cycle Assay Kit (#C1052, Beyotime Biotechnology, Shanghai, China). Cells were resuspended with 500 μL of PI staining solution per tube, incubated at 37°C for 30 min, protected from light, and subjected to flow cytometry (CytoFLEX, BD Biosciences, NY, United States) upon excitation.

### 2.8 Dual luciferase reporter gene assays

PCR amplification was employed to isolate a 400 bp DNA fragment located upstream and downstream of the SNP rs327571319 locus. Subsequently, this fragment was cloned into a PGK vector for the validation of enhancer activity. The constructed plasmid was then transfected into 293 T cells using a transfection reagent (Qiagen). Concurrently, co-transfection involved a plasmid harboring Renilla luciferase (PRL-TK) to serve as an internal control. After 24 h of transfection, luciferase signal transduction was quantified using the dual luciferase reporter assay kit (Vazyme).

### 2.9 Motif analysis

To find the bound transcription factors, transcription factor motifs (TFs) bound to rs327571319 were looked at using TFs from the JASPAR public database.

### 2.10 Statistical analysis

The mRNA expression levels were calculated with the 2-ΔΔCt method and displayed as mean ± standard deviation. Unpaired two-tailed Student’s *t*-test was used to calculate the *p*-value. The t-test was adopted to detect differences between groups for statistical significance. All analytical data were obtained from three independent experiments and each experiment was performed in triplicate.

## 3 Results

### 3.1 *TP63* serves as a candidate regulator of skeletal muscle development

**Figure 1:**
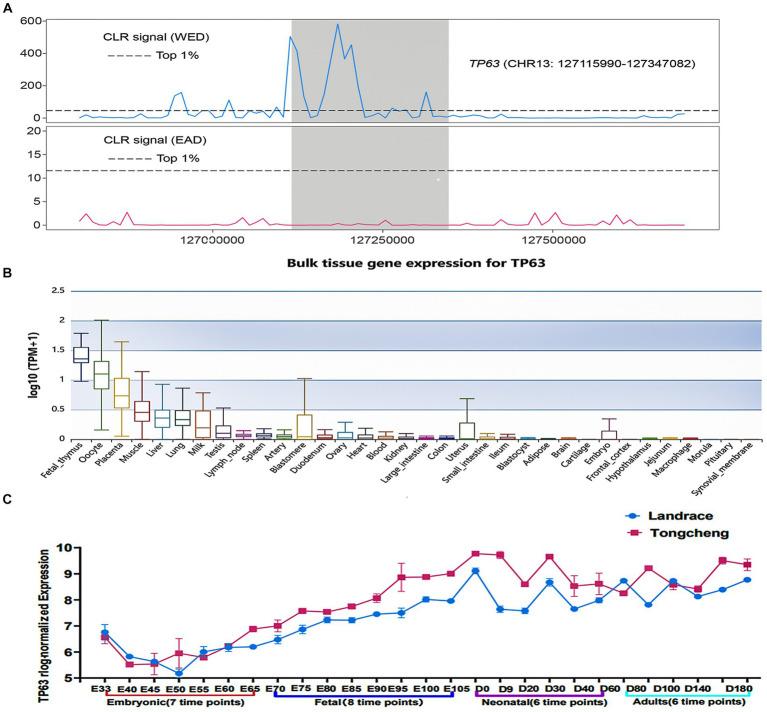
*TP63* is a candidate regulator of skeletal muscle development. **(A)** Selective sweep analysis indicating strong artificial selection acting on *TP63* in western pig breeds (WED) compared to Eastern pigs (EAD). **(B)** Tissue-specific expression of *TP63* in pigs, highlighting its enriched expression in muscle tissues relative to other tissues. **(C)** Differential expression patterns of *TP63* in different developmental stages of skeletal muscle.

The intricate molecular mechanisms governing skeletal muscle development have garnered significant attention due to their impact on muscle development and therapeutic strategies for muscular disorders. Selective sweep analyses demonstrated *TP63* as a highly selected gene in Western lean pig breeds (WED) compared to Eastern pigs (EAD) (Figure 1A), indicating its potential involvement in skeletal muscle development. To validate its tissue-specific expression, we leveraged the Pig GTEx Atlas database, revealing robust *TP63* expression in muscle tissue relative to other tissues (Figure 1B). Additionally, based on our previous RNA-seq data from skeletal muscle at different developmental stages, we examined the expression profiles of *TP63* in Tongcheng and Landrace pigs during various developmental stages of skeletal muscle. We observed a significant disparity in *TP63* expression levels between these two pig breeds, with Tongcheng pigs exhibiting notably higher expression of *TP63* compared to Landrace pigs across fetal, neonatal, and adult stages of skeletal muscle development (Figure 1C). The above findings highlight *TP63* as a promising candidate gene implicated in the regulation of skeletal muscle development.

### 3.2 *TP63* promotes the proliferation of mouse myoblasts

**Figure 2:**
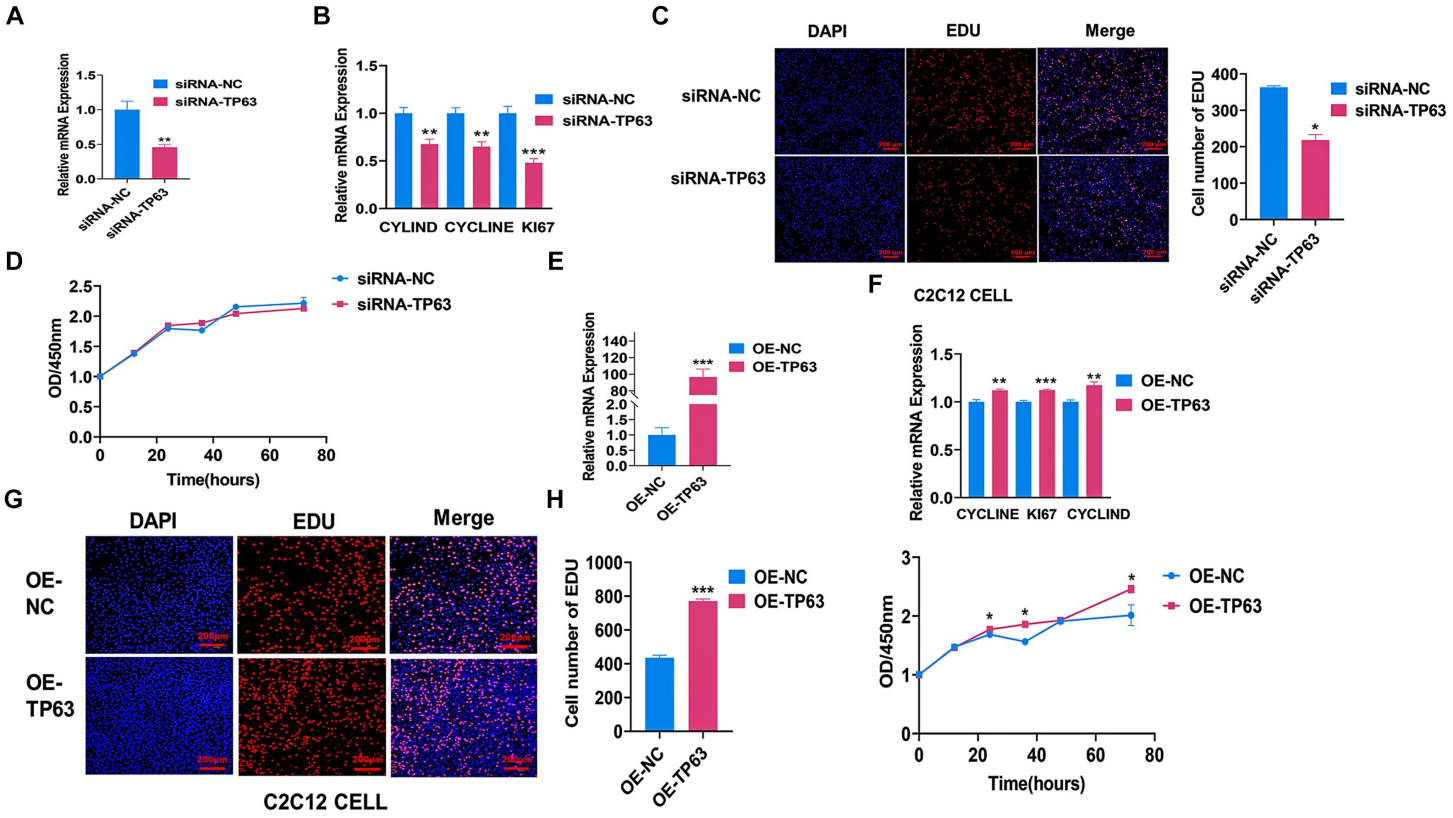
*TP63* promotes the proliferation of C2C12 cells. **(A)** The efficiency of *TP63* knockdown in C2C12 cells was assessed. **(B)** RT-qPCR analysis was performed to determine the mRNA expression levels of proliferation marker genes inhibited by siRNA-*TP63*. **(C)** EdU staining was utilized to detect the proliferative ability of C2C12 cells after *TP63* inhibition. The number of EdU-stained (red) positive cells, DNA-replicating cells, and Hoechst-stained (blue) nuclei were quantified. Scale bar = 200 μm. **(D)** Cell proliferation was assessed using the CCK-8 assay after siRNA-*TP63* transfection in C2C12 cells. **(E)** The efficiency of *TP63* overexpression in C2C12 cells was evaluated. **(F)** RT-qPCR assay was conducted to determine the mRNA expression level of the proliferation marker gene increased by *TP63* overexpression. **(G)** EdU staining was used to detect the proliferation ability of C2C12 cells after *TP63* overexpression. Scale bar = 200 μm. **(H)** Cell proliferation was evaluated using the CCK-8 assay. All experiments were conducted with at least three biological replicates and data were normalized by GAPDH. The results are presented as mean ± SEM (*t*-test). Statistical significance was determined as follows: *p < 0.05, **p < 0.01, ***p < 0.001, *p* ≥ 0.05: ns (not statistically significant).

To investigate the effect of *TP63* on myoblast proliferation, we first utilized mouse C2C12 adult myoblasts and performed functional experiments involving transfection with *TP63* overexpression vectors and siRNA-*TP63* designed to target *TP63*. Our results demonstrated that the knockdown of *TP63* effectively attenuated the mRNA level of *TP63* in mouse C2C12 myoblasts, this knockdown rate is about 60% compared to the control group (Figure 2A), as well as several proliferation marker genes, including *CYCLINE*, *CYCLIND*, and *KI67* (Figure 2B). EdU assay results further revealed a decrease in cell proliferation activity upon *TP63* knockdown (Figure 2C). The CCK8 assay revealed a decline in cell proliferation viability upon *TP63* knockdown, but not significant change. (Figure 2D). Furthermore, the RT-qPCR experiments showed that overexpression of *TP63*, this overexpression efficiency increased about 100-fold compared to the control (Figure 2E) significantly up-regulated the expression of proliferation marker genes (Figure 2F). EdU assays showed an increased number of EdU-positive cells following *TP63* overexpression (Figure 2G), suggestive of enhanced proliferation. Moreover, cell viability assays demonstrated a significant increase in cell viability upon *TP63* overexpression (Figure 2H). The findings indicate that *TP63* enhances the proliferation of murine myoblasts and potentially contributes to the process of myogenesis.

### 3.3 *TP63* promotes the proliferation of porcine skeletal muscle cells

**Figure 3:**
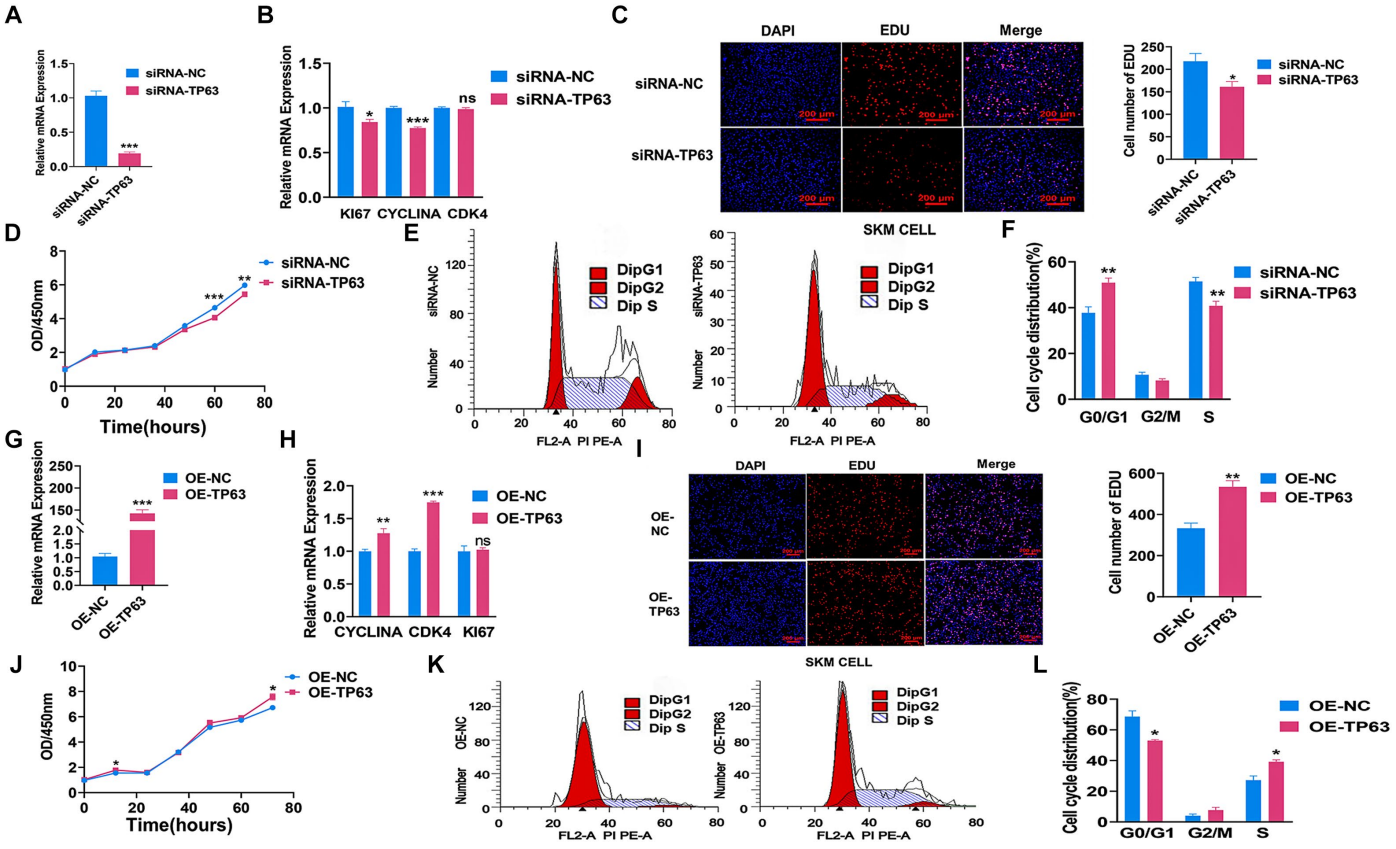
*TP63* regulates the proliferation of porcine skeletal muscle cells. **(A)** The efficiency of *TP63* knockdown in porcine skeletal basal cells was assessed. **(B)** RT-qPCR analysis was conducted to determine the knockdown effect of *TP63* on the mRNA expression levels of cell proliferation marker genes. **(C)** EdU staining was performed to detect the proliferative ability of porcine skeletal muscle cells after *TP63* inhibition. Scale bar = 200 μm. **(D)** Cell proliferation was assessed using the CCK-8 assay after siRNA-*TP63* transfection in porcine skeletal basal cells. **(E,F)** Cell cycle analysis was conducted by flow cytometry after siRNA-*TP63* transfection in proliferating porcine skeletal muscle cells and its negative control. **(G)** The efficiency of *TP63* overexpression in porcine skeletal basal cells was evaluated. **(H)** Overexpression of *TP63* increased the mRNA expression level of cell proliferation marker genes. **(I)** EdU staining was performed to detect the proliferative ability of porcine skeletal muscle cells after *TP63* overexpression. Scale bar = 200 μm. **(J)** Cell proliferation was assessed using the CCK-8 assay after OE-*TP63* transfection in porcine skeletal basal cells. **(K,L)** Cell cycle analysis was conducted by flow cytometry after OE-*TP63* transfection in proliferating porcine skeletal muscle cells and its negative control. All experiments were conducted with at least three biological replicates and data were normalized by GAPDH. The results are presented as mean ± SEM (*t*-test). Statistical significance was determined as follows: *p < 0.05, **p < 0.01, ***p < 0.001, *p* ≥ 0.05: ns (not statistically significant).

To further validate the function of *TP63* in cell proliferation, we performed multiple experiments in the porcine skeletal muscle cells. Our findings demonstrated that siRNA-mediated *TP63* knockdown, The knockdown efficiency reached approximately 80% when compared to the control group, underscoring a significant reduction in target gene expression consequent to the experimental intervention (*p* < 0.01; Figure 3A) and efficiently reduced the mRNA expression levels of critical proliferation marker genes, namely *KI67*, *PCNA*, and *CYCLINA* (Figure 3B). Consistently, *TP63* depletion resulted in a decrease in the number of EdU-positive myoblasts, reflecting diminished proliferative capacity (*p* < 0.05; Figure 3C). Furthermore, the CCK8 assay revealed a significant decline in cell proliferation viability upon *TP63* knockdown (Figure 3D). Remarkably, cell cycle analysis unveiled an altered distribution of cells progressing through the cell cycle, with an increased proportion of cells arrested at the G0/G1 phase and a reduced number of cells transitioning to the S phase (Figures 3E,F). Conversely, *TP63* overexpression in porcine skeletal basal cells. The overexpression efficiency surged approximately 130-fold relative to the control (*p* < 0.001; Figure 3G), leading to a remarkable increase in the mRNA expression levels of *TP63* and key proliferation marker genes (Figure 3H). Intriguingly, both EdU and CCK-8 assays confirmed that *TP63* overexpression significantly enhanced cell proliferative activity (Figures 3I,J). Moreover, cell cycle analysis demonstrated an augmented number of cells progressing to the S phase upon up-regulation of *TP63* (Figures 3K,L). These findings shed light on the essential function of *TP63* in orchestrating the proliferation of porcine skeletal muscle cells, potentially contributing to our understanding of muscle development and regeneration mechanisms.

### 3.4 rs327571319 prioritized as a potential functional SNP

**Figure 4:**
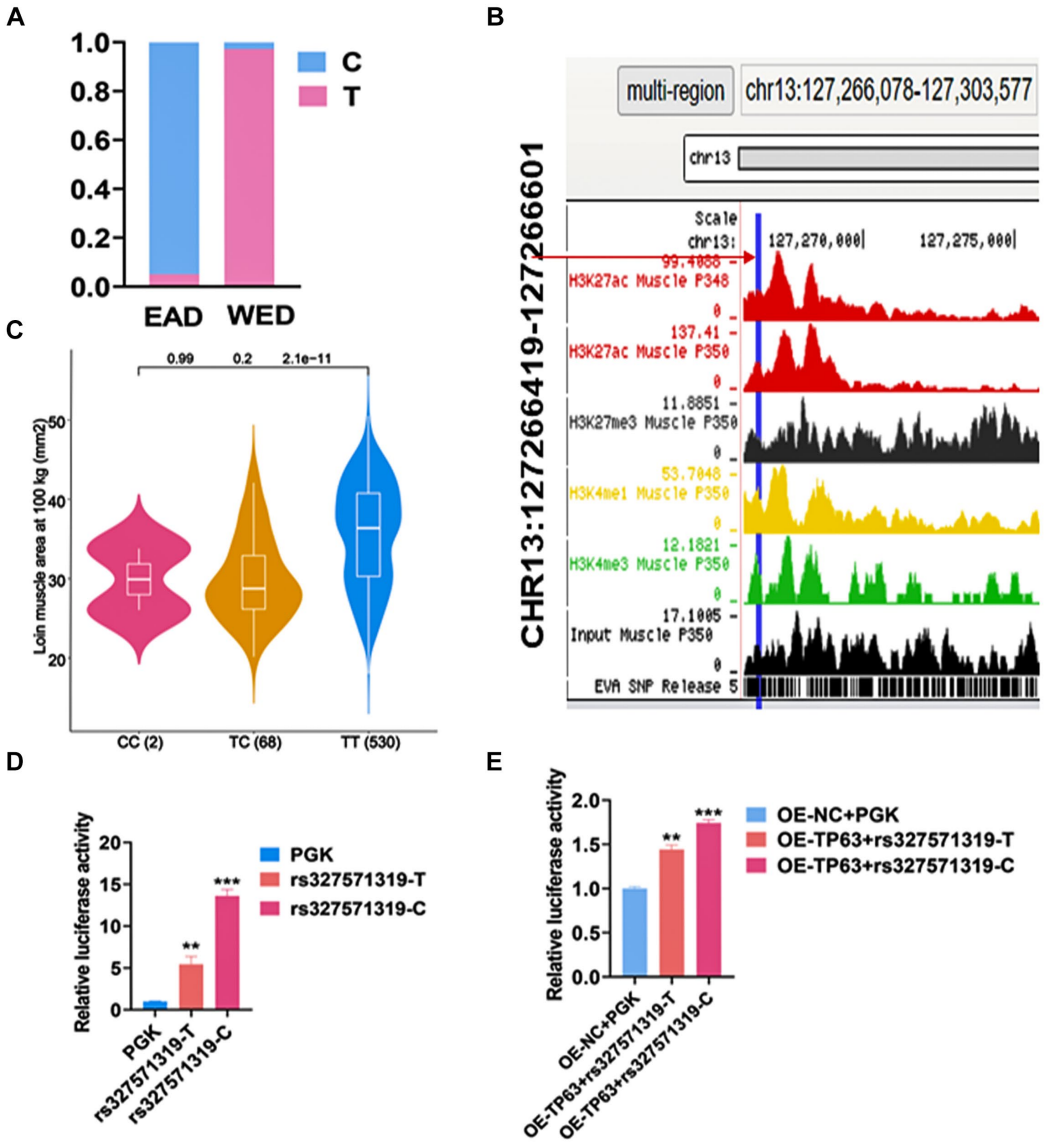
rs327571319 prioritized as a potential functional SNP. **(A)** The gene frequency of the rs327571319 locus indicates a significant difference in allele frequencies between western and eastern pigs. **(B)** Chromatin status data for the rs327571319 locus is presented, suggesting that it may have regulatory significance. **(C)** This SNP was associated with Loin muscle area at 100 kg (LMA). **(D)** Luciferase reporter assays conducted in HEK293T cells compare the enhancer activity between the two alleles of rs327571319, indicating a significant difference in activity. **(E)** In 293 T cells co-transfected with a luciferase reporter plasmid containing the SNP locus and a *TP63* overexpression plasmid, the relative luciferase activity was assayed. All experiments were conducted with at least three biological replicates and data were normalized by GAPDH. The results are presented as mean ± SEM (t-test). Statistical significance was determined as follows: *p < 0.05, **p < 0.01, ***p < 0.001, *p* ≥ 0.05: ns (not statistically significant).

To explore the functional genetic variants regulating *TP63* in myogenesis, we conducted systematic analyses based on public datasets. We utilized genotype files comprising 570 samples from eastern (EAD) population and 296 samples from western pigs (WED) to identify SNPs in the promoter or intron region of *TP63* that exhibited a gene frequency difference greater than 0.9 between the two community from our previous study (18). One candidate SNP, chr13: 127266445 (rs327571319 T/C) located in the fifth intron of *TP63*, stood out as it displayed a T allele frequency of 0.972 in Western pigs and only 0.051 in Eastern pigs (Figure 4A). Chromatin state data showed that the mutation was located in the *TP63* enhancer region (Figure 4B). Further investigation revealed that this SNP was associated with loin muscle area at 100 kg (LMA) (Figure 4C) (31). To determine its functional impact, we performed a dual luciferase assay using vectors containing the wild-type sequence (PGL4.23-rs327571319-T) or the mutated sequence (PGL4.23-rs327571319-C). The results demonstrated that the T-to-C mutation significantly altered enhancer activity (*p* < 0.001; Figure 4D). Moreover, in 293 T cells co-transfected with *TP63* overexpression (*TP63*-OE) along with rs327571319T/C and PRL-TK plasmid, luciferase activity was significantly higher in the presence of OE-*TP63*-rs327571319-C + TK compared to OE-*TP63*-rs327571319-T + TK, confirming the regulatory relationship between *TP63* and rs327571319 (*p* < 0.001; Figure 4E). These findings suggest that rs327571319 could potentially be a functional candidate SNP involved in the regulation of *TP63* and myogenesis.

### 3.5 *MEF2A* binds preferentially to rs327571319(C) to regulate *TP63* expression

**Figure 5:**
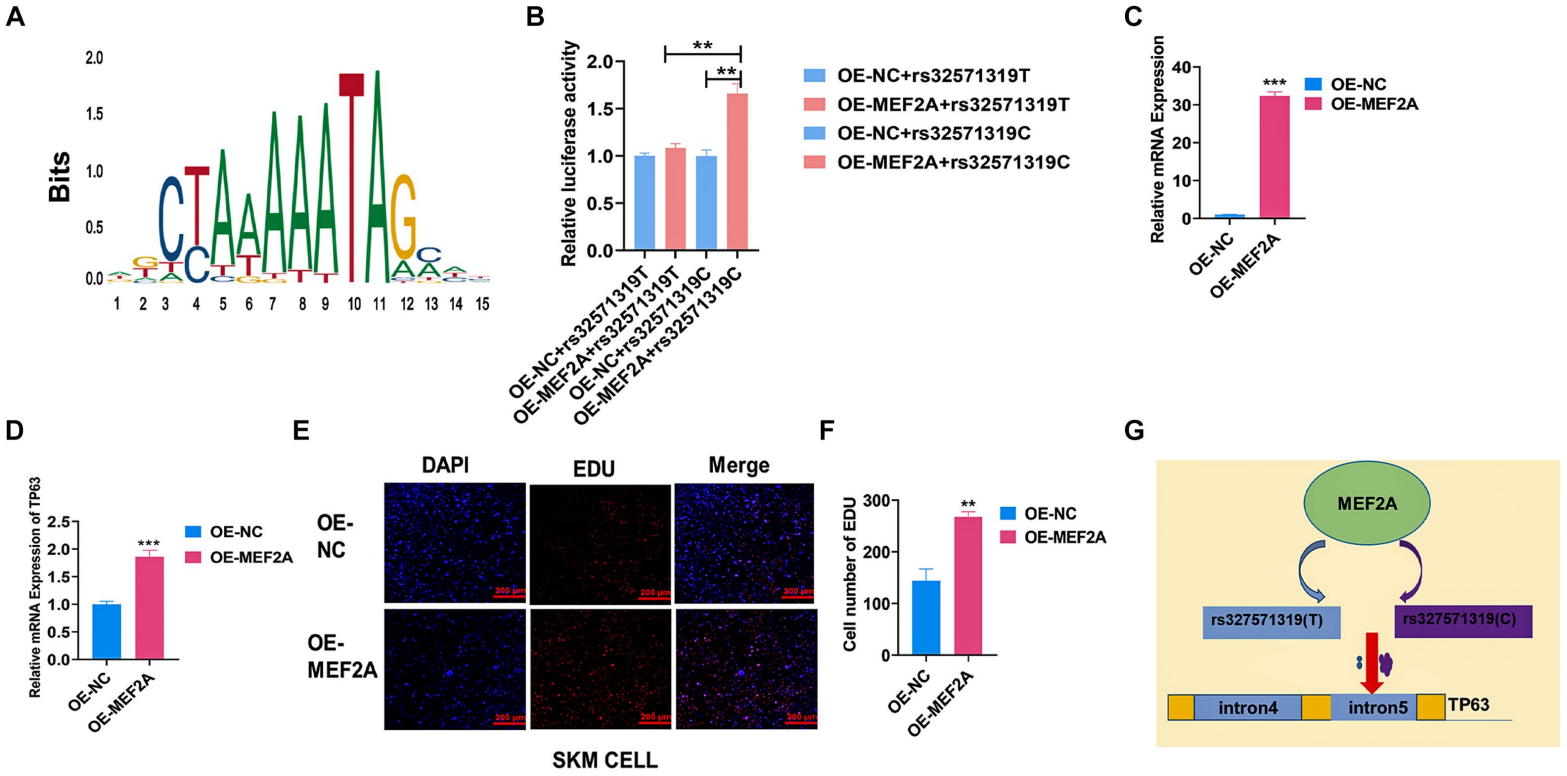
*MEF2A* binds preferentially to rs327571319(C) to regulate *TP63* expression. **(A)** Predicted motif sequences for mutant site binding using the JASPAR database. **(B)** Co-transfection experiments were performed in 293 T cells, with luciferase plasmids containing the SNP site and *MEF2A* overexpression plasmids, to assay relative luciferase activity. **(C)** Efficiency assay of *MEF2A* overexpression in porcine skeletal basal cells. **(D)** Overexpression of *MEF2A* significantly increased the mRNA expression level of *TP63.* **(E,F)** EdU staining assay was conducted to evaluate the proliferative activity of porcine skeletal muscle cells overexpressing *MEF2A*. Scale bar = 200 μm. **(G)** Schematic representation illustrating how the rs327571319 locus affects *TP63* expression. All experiments were conducted with at least three biological replicates and data were normalized by GAPDH. The results are presented as mean ± SEM (*t*-test). Statistical significance was determined as follows: *p < 0.05, **p < 0.01, ***p < 0.001, *p* ≥ 0.05: ns (not statistically significant).

Skeletal muscle development is a highly intricate process governed by the coordinated actions of numerous transcription factors (TFs) and genetic variants. Here, we endeavored to investigate the impact of a putative functional single nucleotide polymorphism (SNP), rs327571319, located within the intronic region of *TP63*, on skeletal muscle development. Through motif analysis using JASPAR, *MEF2A* caught our attention due to its reported promotion of myoblast differentiation (Figure 5A) (32). To elucidate the specific regulatory role of *MEF2A* in relation to the rs327571319 (T/C) allele, we conducted co-transfection experiments by overexpressing *MEF2A* along with plasmids containing the rs327571319-C or rs327571319-T allele in 293 T cells. Co-transfection experiments revealed a significant increase in enhancer activity associated with the rs327571319-C allele, suggesting the greater binding capacity of *MEF2A* on the C allele (*p* < 0.01; Figure 5B). Moreover, overexpression of *MEF2A* in porcine skeletal muscle cells (*p* < 0.001; Figure 5C) resulted in a substantial upregulation of *TP63* expression (*p* < 0.001; Figure 5D). Consistent with these findings, the EdU assay demonstrated an augmented cell proliferative activity (*p* < 0.01; Figures 5E,F). Our study provides evidence that the rs327571319-C allele within the *TP63* intronic region interacts with *MEF2A*, leading to increased *TP63* expression and enhancer activity (Figure 5G). These regulatory changes promote cell proliferation and contribute to the regulation of skeletal muscle development.

## 4 Discussion

The aim of this study was to explore the regulatory mechanism of *TP63* in porcine skeletal muscle cells. Skeletal muscle development is a crucial process in animal growth and development, in which the proliferation, differentiation, and fusion of satellite cells play a pivotal role in this process (33). Selective sweep analyses have identified *TP63* as a highly selected gene in Western lean pig breeds compared to Eastern pigs, drawing our attention to its potential role in skeletal muscle development. Its role in porcine skeletal muscle development remains largely unexplored. Our study provides novel insights into the regulatory mechanisms underlying porcine skeletal muscle development.

Previous studies have shown that *TP63* plays diverse roles in different cell types, including promoting cell proliferation and regulating late stages of myogenic differentiation (34). For instance, *TP63* has been shown to promote the proliferation of esophageal squamous cell carcinoma (ESCC) cells by binding to the enhancer on the LINC01503 motif (35, 36). Additionally, *TP63* has been reported to function at a late stage of myogenic differentiation (37), affecting the expression of genes associated with myogenesis and skeletal muscle contractility upon knockdown of TAp63γ (38). However, Li et al. reported that inhibition of *TP63* expression promotes ciliated cells (39). Our results showed that *TP63* positively correlated with skeletal muscle cell proliferation. These findings enhance our understanding of the factors influencing muscle development and shed light on the involvement of *TP63* in skeletal muscle cell proliferation.

In the context of active euchromatin, transcription factors (TFs) exhibit the capacity to bind open chromatin regions harboring DNA regulatory elements, thereby modulating gene expression (40). Recent investigations have unveiled the pivotal regulatory role of *TP63* in orchestrating chromatin accessibility and enhancer reprogramming in keratin-forming cells (41, 42). The functional significance of p63-binding enhancers is underscored by their correlation with the dynamics of gene expression, indicative of *TP63*’s crucial involvement in gene regulation through enhancer activation (42). Our investigation has delineated the functional implication of *MEF2A* binding to a functional SNP, rs327571319-C, which serves as a critical modulator of enhancer activity and *TP63* expression. This regulatory axis exerts influence over cellular proliferation, shedding light on the intricate regulatory mechanisms governing *TP63* expression and its downstream effects on cellular processes relevant to meat production traits. Notably, previous RNA-seq data analysis has revealed differential expression patterns of *TP63* in lean-type Landrace pigs compared to obese-type Tongcheng pigs, aligning with the observed frequency distribution of the rs327571319-T allele in Western pig populations. This observation underscores the potential influence of *TP63* on meat production traits and positions it as a promising candidate for utilization as a breeding marker, offering avenues for genetic improvement in the context of meat production.

Myocyte enhancer factor 2 (MEF2) is a member of the MADS superfamily of transcription factors that play a crucial role in regulating muscle-specific gene expression (43). Previous studies have demonstrated the involvement of *MEF2A* in promoting cardiomyocyte proliferation and adult myocyte proliferation (44, 45). Our findings indicate that *MEF2A* also promotes skeletal muscle cell proliferation, further expanding our understanding of its regulatory functions in muscle development. However, the current study is limited by the absence of *in vivo* functional validation experiments, as well as the lack of investigation into muscle regeneration and the effects of *TP63* and *MEF2A* knockdown in mice. Future studies will be conducted to address these gaps and provide a more comprehensive understanding of the mechanisms underlying myogenesis.

## 5 Conclusion

In summary, our study provides valuable insights into the regulatory factors involved in skeletal muscle development and highlights the involvement of *TP63* and *MEF2A* in the proliferation and differentiation of skeletal muscle cells. These findings provide a foundation for further exploration of *TP63*’s role in shaping phenotypic traits relevant to meat production and offer potential avenues for genetic enhancement in the pig industry.

## Data availability statement

The original contributions presented in the study are included in the article/Supplementary material, further inquiries can be directed to the corresponding authors.

## Ethics statement

Ethical approval was not required for the studies on animals in accordance with the local legislation and institutional requirements because only commercially available established cell lines were used.

## Author contributions

YC: Data curation, Validation, Writing – original draft, Writing – review & editing. ZW: Formal analysis, Validation, Writing – original draft. XQ: Data curation, Formal analysis, Writing – original draft. BS: Data curation, Software, Validation, Writing – original draft. YT: Data curation, Software, Validation, Writing – original draft. BL: Writing – review & editing. GC: Writing – review & editing. GY: Conceptualization, Funding acquisition, Writing – review & editing.
