# Supplementary material for: An intronic SNP affects skeletal muscle development by regulating the expression of TP63
Source: Front Vet Sci. 2024 Jun 12;11:1396766. doi: 10.3389/fvets.2024.1396766 (PMC11199888; doi:10.3389/fvets.2024.1396766)
Supplement: Supplementary file 1 [file Table_1.docx]

| **Table S1.** The sequences of siRNA | |  |
| --- | --- | --- |
| **Name** | **Sequence** | **Tm (℃)** |
| *SiRNA-NC* | F: UUCUCCGAACGUGUCACGUTT | 60 |
|  | R: ACGUGACAAGUUCGGAGAATT |  |
| *SiRNA-TP63* | F: CCUCAGCACACGAUCGAAATT | 55.9 |
|  | R: UUUCGAUCGUGUGCUGAGGTT |  |

|  | **Table S2.** The primers information used in the RT-Qpcr（mouse） | | |
| --- | --- | --- | --- |
| **Gene name** | **Primer Sequence (5’-3’)** | **Tm (℃)** | **Product Size (bp)** |
| *GAPDH* | F: CCTGTTGCTGTAGCCGTATT | 54 | 138 |
|  | R: CATCAAGAAGGTGGTGAAGC |  |  |
| *TP63* | F: ATGAGCTGAGCCGTGAGTTCAA | 57 | 125 |
|  | R: TACAGGACTGTTGTGAATTCA |  |  |
| *KI67* | F: ATCATTGACCGCTCCTTTAGGT | 57 | 160 |
|  | R: GCTCGCCTTGATGGTTCCT |  |  |
| *PCNA* | F: GGGTGAAGTTTTCTGCAAGTG | 55 | 141 |
|  | R: GTACCTCAGAGCAAACGTTAGG |  |  |
| *CYCLINE* | F: GCGAGGATGAGAGCAGTTC | 56 | 120 |
|  | R: AAGTCCTGTGCCAAGTAGAAC |  |  |

|  | **Table S3. The primers information used in the RT-Qpcr（pig）** | | |
| --- | --- | --- | --- |
| ***Gene name*** | P**rimer Sequence (5’-3’)** | **Tm (℃)** | **Product Size (bp)** |
| *β-actin* | F: GCGGCATCCACGAAACTAC | *60* | *138* |
|  | R: TGATCTCCTTCTCATCCTGTC |  |  |
| *TP63* | F: ATGCCTGTCTACAAGAAGGCT | *57* | *130* |
|  | R: CACGCTCTGTCTTTCCTGTGA |  |  |
| *KI67* | F: AGCCCGTATCTGGTGCAAAA | *60* | *267* |
|  | R: CCTGCATCTGTGTAAGGGCA |  |  |
| *PCNA* | F: AAGTCAAATCTGGTCTGTTAGCC | *60* | *141* |
|  | R: CACTGTCCTGGGATGCTTGAA |  |  |
| *CDK4* | F: TGGTTACAAGTGGTGGGACA | *59* | *111* |
|  | R: CTGGAGCACGGTACCAGAGT |  |  |
| *CYCLINA* | F: GCAGCAGCCTTTCATTTAGC | *58* | *118* |
|  | R: GGTGAAGGTCCAGGAGACAA |  |  |
| *MEF2A* | F: CTCAGAGACCACCAAGTCA | *54* | *158* |
|  | R: GACAACGCGAAGATCTGGTT |  |  |
